# Supplementary material for: Azospirillum brasilense and Zinc Rates Effect on Fungal Root Colonization and Yield of Wheat-Maize in Tropical Savannah Conditions
Source: Plants (Basel). 2022 Nov 18;11(22):3154. doi: 10.3390/plants11223154 (PMC9694232; doi:10.3390/plants11223154)
Supplement: Supplementary file 1 [file plants-11-03154-s001.zip › plants-1972533-supplementary.pdf]

Azospirillum brasilense and zinc rates effect on fungal root colonization and yield of wheat-maize in tropical savannah conditions

Philippe Solano Toledo Silva[1], Ana Maria Rodrigues Cassiolato[2], Fernando Shintate Galindo[3], Arshad Jalal[2], Thiago Assis Rodrigues Nogueira[2], Carlos Eduardo da Silva Oliveira[2] and Marcelo Carvalho Minhoto Teixeira Filho[2]*

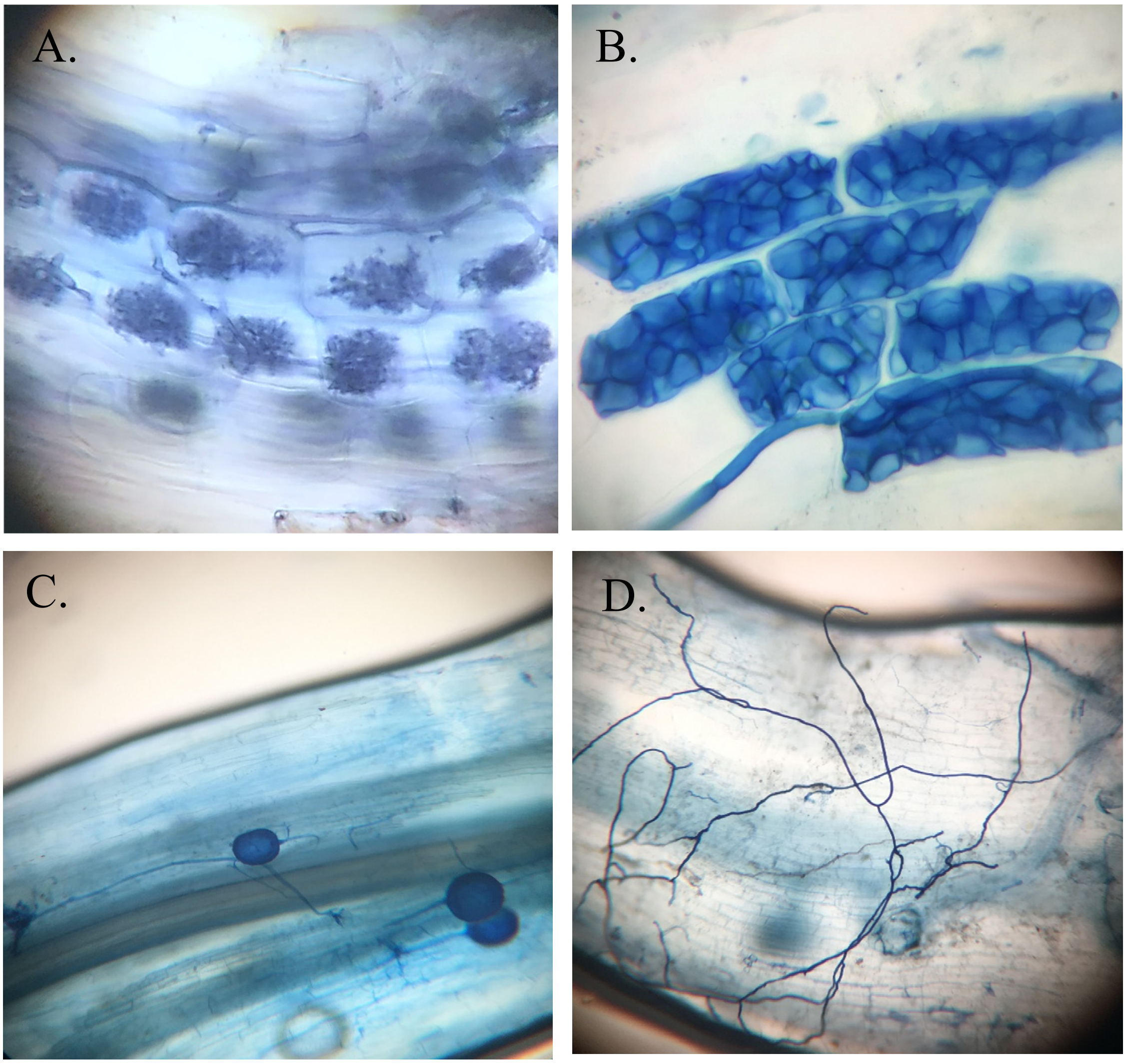

Supplementary Figure S1. Hyphae and arbuscules of arbuscular mycorrhizal fungus (A), hyphae and microsclerotia of melanized septate endophytic fungus in corn roots (B). Hyphae and vesicles of arbuscular mycorrhizal fungus (C) and melanized septate hyphae on wheat roots (D).

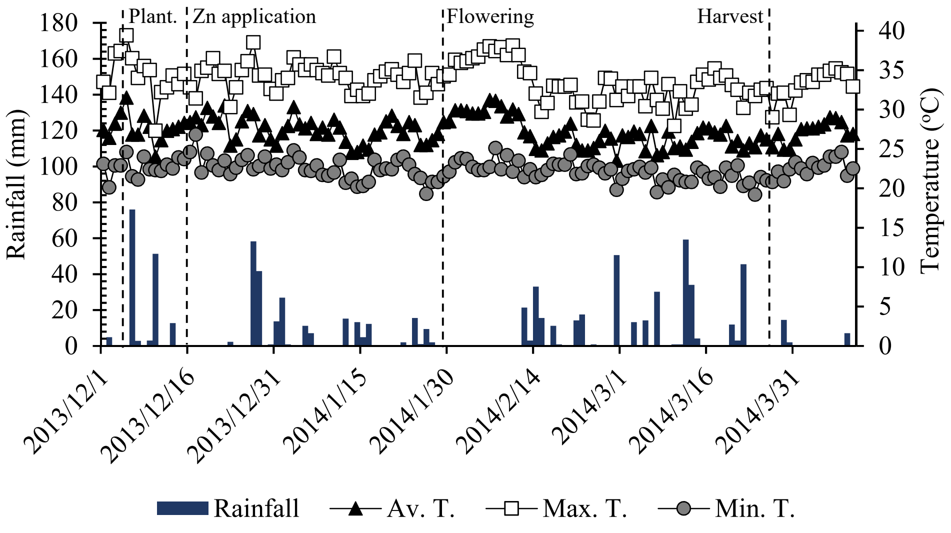

(A)

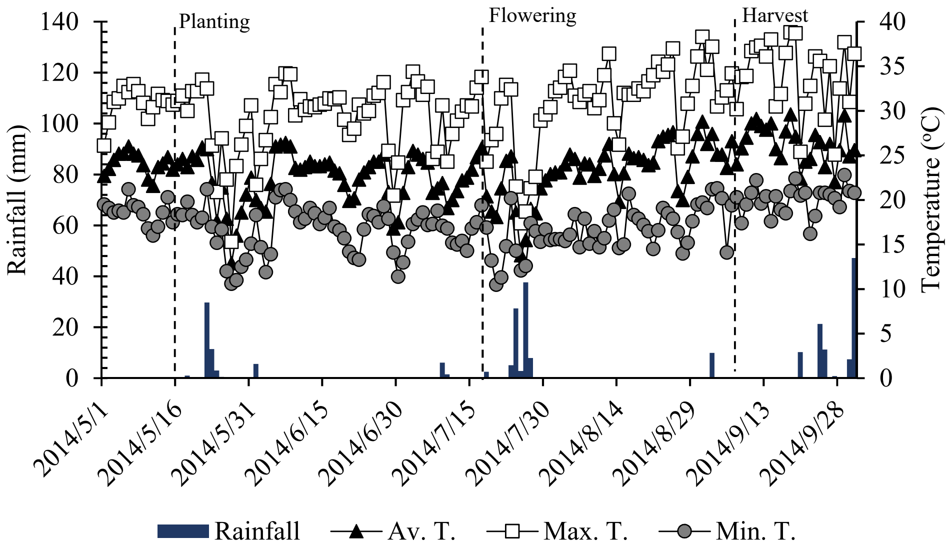

(B)

Supplementary Figure S2. Rainfall, maximum, average and minimum temperatures obtained from the weather station located in the Education and Research Farm of FE / UNESP during the maize (A) and wheat (B) cultivation.
